# Measures of harm reduction service provision for people who inject drugs

**DOI:** 10.2471/BLT.18.224089

**Published:** 2019-06-20

**Authors:** Daniel O’Keefe, Ricky N Bluthenthal, Alex H Kral, Campbell K Aitken, Angus McCormack, Paul M Dietze

**Affiliations:** a Behaviours and Health Risks, Burnet Institute, 85 Commercial Rd, Melbourne, Victoria, 3004, Australia.; b Institute for Health Promotion and Disease Prevention, University of Southern California, Los Angeles, United States of America (USA).; c Behavioural Health Research Division, RTI International, San Francisco, USA.

## Abstract

Coverage is an important dimension in measuring the effectiveness of needle and syringe programmes in providing sterile injecting equipment for people who inject drugs. The World Health Organization (WHO), the United Nations Office on Drugs and Crime (UNODC) and the Joint United Nations Programme on HIV/AIDS (UNAIDS) currently recommend methods for measuring coverage at the population level, that is, across an estimated population of people who inject drugs within a given geographical area. However, population-level measures of coverage rely on highly uncertain population estimates and cannot capture the different levels of syringe acquisition and injecting episodes among individual users. Consequently, such measures only broadly evaluate the extent of programme service delivery, rather than describe how people who inject drugs as individuals and sub-groups interact with needle and syringe programmes. In response to these limitations, several researchers have proposed measuring coverage at the individual level, by the percentage of injecting episodes in relation to the number of sterile needles and syringes acquired. These measures evaluate coverage according to each individual’s needs. Such measures provide enhanced information for planning and monitoring of harm reduction programmes and have now been used in multiple international research studies. We advise that WHO, UNODC and UNAIDS add individual-level coverage measurement methods to their international monitoring guidelines for harm reduction programmes. By doing this, more responsive and effective programmes can be created to better reduce injecting risk behaviours and blood-borne virus transmission among people who inject drugs.

## Résumé

La couverture est une dimension importante lorsque l'on veut mesurer l'efficacité des programmes de distribution d'aiguilles et de seringues à fournir du matériel d'injection stérile aux consommateurs de drogues par injection. L'Organisation mondiale de la Santé (OMS), l'Office des Nations Unies contre la drogue et le crime (ONUDC) et le Programme commun des Nations Unies sur le VIH/sida (ONUSIDA) recommandent actuellement des méthodes pour mesurer la couverture au niveau de la population, c'est-à-dire sur une population estimée de consommateurs de drogues par injection dans une zone géographique donnée. Or, les mesures de la couverture au niveau de la population se fondent sur des estimations très incertaines de la population et ne permettent pas de refléter les différents degrés d'acquisition de seringues et d'épisodes d'injection chez les usagers. Par conséquent, ces mesures n'évaluent que globalement la portée des programmes au lieu de décrire la manière dont les consommateurs de drogues par injection interagissent, individuellement et en sous-groupes, avec les programmes de distribution d'aiguilles et de seringues. En réponse à ces limitations, plusieurs chercheurs ont proposé de mesurer la couverture au niveau individuel, en calculant le pourcentage d'épisodes d'injection par rapport au nombre d'aiguilles et de seringues stériles acquises. Ces mesures permettent d'évaluer la couverture en fonction des besoins de chaque personne. Ce type de mesures offre des informations plus fiables pour la planification et le suivi des programmes de réduction des risques et il est aujourd'hui utilisé dans plusieurs études de recherche internationales. Nous suggérons à l'OMS, à l'ONUDC et à l'ONUSIDA d'ajouter des méthodes de mesure de la couverture au niveau individuel à leurs directives internationales pour le suivi des programmes de réduction des risques. Cela permettra de mettre au point des programmes plus adaptés et efficaces afin de mieux réduire les comportements à risque liés aux injections ainsi que la transmission de virus par le sang chez les consommateurs de drogues par injection.

## Resumen

La cobertura es un factor importante para medir la eficacia de los programas de agujas y jeringas en el suministro de equipo de inyección estéril para las personas que se inyectan drogas. La Organización Mundial de la Salud (OMS), la Oficina de las Naciones Unidas contra la Droga y el Delito (ONUDD) y el Programa Conjunto de las Naciones Unidas sobre el VIH/SIDA (ONUSIDA) recomiendan actualmente métodos para medir la cobertura a nivel poblacional, es decir, a través de una población estimada de consumidores de drogas inyectables dentro de una zona geográfica determinada. Sin embargo, las medidas de cobertura a nivel poblacional se basan en estimaciones poblacionales altamente inciertas y no pueden captar los diferentes niveles de adquisición de jeringas y episodios de inyección entre los usuarios individuales. En consecuencia, esas medidas solo miden en términos generales el alcance de la prestación de servicios de los programas, en lugar de describir la forma en que las personas que se inyectan drogas como individuos y subgrupos interactúan con los programas de suministro de agujas y jeringas. En respuesta a estas limitaciones, varios investigadores han propuesto medir la cobertura a nivel individual, por el porcentaje de episodios de inyección en relación con el número de agujas y jeringas estériles adquiridas. Estas medidas miden la cobertura de acuerdo a las necesidades de cada individuo. Estas medidas proporcionan una mejor información para la planificación y el seguimiento de los programas de reducción de daños y se han utilizado actualmente en múltiples estudios de investigación internacionales. Aconsejamos que la OMS, la ONUDD y el ONUSIDA incorporen métodos de medición de la cobertura a nivel individual a sus directrices internacionales de vigilancia de los programas de reducción de daños. De este modo, se pueden crear programas más receptivos y eficaces para reducir mejor los comportamientos de riesgo en el uso de drogas inyectables y la transmisión de virus transmitidos por la sangre entre las personas que se inyectan drogas.

## ملخص

تعتبر التغطية بعداً هاماً في قياس فعالية برامج الإبر والمحاقن في توفير معدات الحقن المعقمة للأشخاص الذين يتعاطون المخدرات. ****إن كل من منظمة الصحة العالمية (WHO)، ومكتب الأمم المتحدة للمخدرات والجريمة (UNODC)، وبرنامج الأمم المتحدة المشترك الخاص بفيروس العوز المناعي/الإيدز (UNAIDS)، توصي حالياً بطرق لقياس التغطية على مستوى السكان، أي من خلال عدد السكان التقديري للأشخاص الذين يتعاطون المخدرات في إطار منطقة جغرافية محددة. ****ومع ذلك، تعتمد تدابير التغطية على مستوى السكان على تقديرات سكانية غير مؤكدة تماماً، ولا يمكنها التقاط المستويات المختلفة للحصول على المحاقن وحالات الحقن بين المستخدمين الأفراد. ****وتبعاً لذلك، فإن مثل هذه التدابير تقيّم فقط على نطاق واسع مدى تقديم خدمة البرنامج، بدلاً من وصف كيفية تفاعل الأشخاص الذين يتعاطون المخدرات كأفراد ومجموعات فرعية مع برامج الإبر والمحاقن. ****واستجابة لهذه القيود، اقترح العديد من الباحثين قياس التغطية على المستوى الفردي، عن طريق النسبة المئوية لحالات الحقن بالنسبة لعدد الإبر والحقن المعقمة المطلوبة. ****تقوم هذه التدابير بتقييم التغطية وفقا لاحتياجات كل فرد. ****توفر هذه التدابير معلومات معززة للتخطيط لبرامج الحد من الضرر ومراقبتها، وقد تم استخدامها الآن في العديد من الدراسات البحثية الدولية. ****نحن ننصح بأن تقوم كل من منظمة الصحة العالمية (WHO)، ومكتب الأمم المتحدة للمخدرات والجريمة (UNODC)، وبرنامج الأمم المتحدة المشترك الخاص بفيروس العوز المناعي/الإيدز (UNAIDS)، بإضافة طرق لقياس التغطية على المستوى الفردي، إلى مبادئها التوجيهية الدولية لمراقبة برامج الحد من الضرر. ****ومن خلال القيام بذلك، يمكن وضع برامج أكثر استجابة وفعالية للحد بشكل أفضل من سلوكيات مخاطر الحقن، وانتقال الفيروس عن طريق الدم بين الأشخاص الذين يتعاطون المخدرات.

## 摘要

为注射吸毒者提供无菌注射设备时，覆盖率是衡量注射针头和注射器方案是否有效的重要因素之一。世界卫生组织 (WHO)、联合国毒品和犯罪问题办公室 (UNODC) 和联合国艾滋病毒/艾滋病联合规划署 (UNAIDS) 目前建议采用基于人口的方法来测量覆盖率，即在指定的地理区域内估计注射毒品的人口数量。然而，基于人口水平的覆盖率测量方法高度依赖不确定的预估人口数量，并且无法捕捉到个体使用者获取注射器和注射阶段的不同水平。因此，这些措施只能宽泛地评估此服务方案普及的范围，并不能反映注射毒品的个人和小团体是如何接触使用注射针头和注射器的。为了解决此类限制，一些研究人员建议，以个人为单位，根据注射阶段与获得的无菌注射针头和注射器数量的百分比来测量覆盖率。这些措施会根据个体的需求情况评估覆盖率，为减少伤害方案的规划和监测提供了增强信息，现已在多项国际性研究中得以使用。我们建议世界卫生组织、联合国毒品和犯罪问题办公室和联合国艾滋病毒/艾滋病联合规划署在减少伤害方案的国际监测准则中增加测量个人覆盖率的方法。这样的话，就可以制定出更具反响性、更有效的方案，从而更好地减少注射吸毒者之间的注射风险行为和血源性病毒的传播。

## Резюме

В оценке эффективности программ по распространению игл и шприцев, направленных на обеспечение стерильного инъекционного оборудования людям, принимающим наркотики путем инъекций, важным параметром является степень охвата такими программами. Всемирная организация здравоохранения (ВОЗ), Управление Организации Объединенных Наций по наркотикам и преступности (ЮНОДК) и Объединенная программа Организации Объединенных Наций по ВИЧ/СПИДу (ЮНЭЙДС) в настоящее время рекомендуют использование методов измерения охвата на уровне населения, то есть в рамках оцениваемой в данном географическом регионе популяции лиц, принимающих наркотики путем инъекций. Однако любые попытки измерения охвата программой на уровне населения опираются на весьма неопределенные оценки численности населения и не способны вычленять различные уровни распространения шприцев и случаев введения наркотиков среди отдельных пользователей. Следовательно, подобные измерения способны только поверхностно оценить степень предоставления услуг по программе, но не в состоянии описать, каким образом люди и подгруппы населения, принимающие наркотики путем инъекций, взаимодействуют с программой распространения игл и шприцев. В ответ на эти ограничения некоторые исследователи предложили измерять охват программой на индивидуальном уровне по проценту случаев введения наркотиков в зависимости от количества полученных стерильных игл и шприцев. Эти измеряемые показатели оценивают охват программой в соответствии с потребностями каждого отдельного лица. Такие измеряемые показатели обеспечивают расширенную информацию, необходимую для планирования и мониторинга программ по уменьшению вреда, и теперь они используются во многих международных исследованиях. Авторы рекомендуют ВОЗ, ЮНОДК и ЮНЭЙДС добавить методы измерения охвата программой на индивидуальном уровне в соответствующие международные рекомендации по программам уменьшения вреда. Таким образом можно будет создать более эффективные и реагирующие на изменения ситуации программы, что позволит эффективнее снижать риски, связанные с инъекциями, и уменьшать передачу вирусов с кровью среди лиц, принимающих наркотики путем инъекций.

## Introduction

Coverage is an important concept in the evaluation of any public health intervention and coverage has been defined as “the proportion of the population at risk reached by an intervention, ideally with sufficient intensity to have probable impact.”^1^ Historically associated with communicable disease control and immunization programmes,^2^ coverage provides a means of assessing programmatic effectiveness and developing performance targets. However, the way in which coverage is defined and measured has a direct bearing on estimates of programme outcomes.

Needle and syringe programmes provide sterile injection equipment to people who inject drugs. The aim is to reduce transmission of blood-borne viruses, such as human immunodeficiency virus (HIV) and hepatitis B and C virus (HCV) via the sharing of used syringes.^3^ Such programmes can include dispensing at fixed or mobile sites, via pharmacy sales, from syringe vending machines and peer distribution. Programmes often provide ancillary public health services, such as pathways into drug and alcohol treatment, psychosocial support and provision of naloxone for opioid users. Coverage of needle and syringe distribution is an important dimension in measuring the effectiveness of programmes, although there are several limitations. The ambiguities around the definition, measurement and evaluation of needle and syringe programme coverage have been described.^2^ Importantly, syringe coverage can be measured at the population level or the individual level and these levels suit specific purposes in programmatic monitoring.^2^

The measurement of syringe coverage needs to account for differences among people who inject drugs in terms of barriers to accessing services and individuals’ risk profiles.^4^^–^^6^ High coverage may not be a measure of success if only those with the least risk are covered. How best to identify sub-groups with different risk profiles remains a challenge when measuring coverage. Population-level measurements of coverage are currently recommended by the World Health Organization (WHO), the United Nations Office on Drugs and Crime (UNODC) and the Joint United Nations Programme on HIV/AIDS (UNAIDS).^7^ Although important for monitoring ongoing trends in regional and national service provision, these tools are not intended to detect the unique risks experienced by individual people who inject drugs or to understand the contexts in which drug use occurs. In response, previous researchers have defined coverage in terms of the relationship between an individual’s injecting frequency and his or her syringe acquisition.^8^ Despite the advantages of this method of measuring coverage, which aims to fill gaps in population-level coverage, the method has not been adopted as a standard indicator of programme effectiveness in the same way as population-level measures.

In this paper we explore the development of syringe programme coverage measurement at the individual level, its previous international implementation and the practical considerations and limitations of its use. Ultimately, we argue that individual-level coverage should be considered by the WHO, UNODC and UNAIDS as a complementary indicator for monitoring and planning to provide essential programme evaluation information that population-level measures are unable to fully capture.

## Barriers to programme access

The evidence for the effectiveness of needle and syringe programmes is strong,^9^ but this effectiveness is dependent upon consistently high syringe coverage, especially among those people who inject drugs who are most at risk of disease transmission. However, only a minority of countries with needle and syringe programmes reach the level of syringe distribution recommended by WHO, UNODC and UNAIDS.^7^^,^^10^ Of the 75 countries or territories with data permitting estimation of population-level syringe coverage, only nine reach the benchmark for high coverage: Australia, Austria, Estonia, Finland, Kyrgyzstan, Netherlands, Norway, Scotland and Tajikistan.^10^

Numerous factors affect access to syringe exchange services and therefore coverage, in terms of what services can provide and individuals can achieve.^11^ These factors can be broadly classified as institutional (e.g. government policy),^12^ environmental (e.g. few or difficult to reach needle and syringe programmes)^13^ and individual barriers (e.g. different injecting frequencies, different drug preferences).^5^^,^^14^^,^^15^ Multiple barriers may exist in a given location and may be beyond the control of people who inject drugs as individuals.

Crucially, these barriers do not affect locations, services or individuals in a uniform way, but are context-dependent. For example, poor overall geographical access to needle and syringe programmes will not affect individuals living close to programme sites. There is also substantial international variation in a programme’s capacity to deliver adequate service.^16^ While the barriers to service use and provision, and therefore coverage, cannot be eliminated entirely, the more they can be planned for and mitigated, the greater the potential to maximize coverage.

## Population-level coverage

WHO, UNODC and UNAIDS released the first *Technical guide for countries to set targets for universal HIV services of injecting drug users* in 2009,^17^ and an update in 2012.^7^ The guide specifies methods of measuring coverage as a means of evaluating programme delivery. The guide included previously used measures of reach (the percentage of an estimated population of people who inject drugs regularly, i.e. once per month, interacting with a service) and a refined, updated measure of need (the number of sterile syringes distributed per person across an estimated population of people who inject drugs).^7^ Along with other indicators of effectiveness within the harm reduction package, the guide endorsed these previously used^18^^,^^19^ methods of measuring syringe coverage as standard and accepted means of international harm reduction monitoring and evaluation. In practical terms, the measure of need is considered as the priority indicator, while the measure of reach is now considered an add-on to inform programme monitoring. Due to its importance, the measure of need is a key component of the UNAIDS global acquired immune deficiency syndrome response progress reporting package,^20^ and is considered a means of monitoring the overall disease preventive programme via needle and syringe programmes. The measures can be used to assess the progress of an intervention over time at the regional, national, sub-national and service-delivery levels,^7^ and have been used to compare programme performance across countries.^10^ These measures are intended to assess the delivery and use of services across the entire population of people who inject drugs, thereby describing large-scale programme outcomes and service delivery trends over time.

The goal of needle and syringe programmes is to ensure one sterile syringe is used per injection.^8^ Nevertheless, WHO, UNODC and UNAIDS targets recognize that this goal is aspirational and classify high coverage targets according to modelling studies and expert opinion of real-world outcomes.^21^^,^^22^ The targets define high coverage as 60% of the estimated population of people who inject drugs being regularly reached by services and distribution of ≥ 200 sterile syringes per person per annum, again across the estimated population.^7^ However, these targets may have an inadequate impact on blood-borne virus transmission, a point seemingly acknowledged by WHO, which has recommended as part of its strategy to eliminate global HCV, to increase distribution to 300 syringes per person per annum.^23^ Such a target may still be inadequate for many people who inject drugs.

As standard monitoring and evaluation indicators, these population-level methods and targets are widely recognized and used.^10^^,^^24^ The methods have obvious limitations, however. The measures of both reach and need rely on estimates of the population of people who inject drugs, which are always uncertain.^25^^,^^26^ The measure of reach is reliant on system-wide registration to capture repeat visits by clients,^26^ a practice common to some countries^2^ but not all. Moreover, these overall targets fail to account for the often significant variability in acquisition of syringes, frequency of injecting and ability to access services among people who inject drugs.^25^ For example, many countries may not be able to include pharmacy sales within their calculations of per capita syringe distribution.^7^ Consequently, population-level coverage measures only broadly evaluate the extent of needle and syringe programme service delivery according to a specified target.^2^ These measures cannot, however, provide a better understanding of how people who inject drugs as individuals and groups actually interact with needle and syringe programmes. The distinctions between individual access levels to needle and syringe programmes represents a bias that needs to be considered.^11^

## Individual-level coverage

An individual-level measure of coverage calculates the percentage of injecting episodes covered by the acquisition of a sterile syringe^8^ for each person who injects drugs. In this way, the measure addresses the multifactorial differences between people and provides a broader picture of the facilitators and barriers to service use than simply the extent of service delivery.

Formalized methods of calculating individual-level coverage are relatively recent. The most well-known of these methods was developed in 2007.^8^ Using primary data collection, the researchers recorded for each individual the number of syringes retained (i.e. syringes acquired, minus those intended to be distributed or already given away) at the last needle and syringe programme visit. The number of syringes retained is multiplied by the number of needle and syringe programme visits in the past 30 days, and then divided by the self-reported injecting frequency within the same time period.^8^ Accordingly, ≥ 100% coverage suggests that all injections were covered by at least one acquired sterile syringe and that coverage was therefore sufficient for that individual. The prevalence of sufficient or insufficient individual-level coverage among the sample can then be estimated.

By recording differential syringe acquisition and injecting frequency, individual-level coverage measurement accounts for the cluster of behaviours associated with, and influencing, syringe acquisition and use. For example, the measure can account for secondary exchange of previously acquired syringes with other people who inject drugs.^27^^,^^28^ Also, because individual-level measures necessitate primary data collection, demographic and behavioural exposure variables can be collected at the same time and can then be tested for associations with coverage outcomes.^8^^,^^29^^,^^30^

## Research evidence

The original work describing the individual-level coverage measure found an inverse relationship between individual-level syringe coverage and injecting risk: the lower the coverage, the higher the percentage of those displaying injecting risk behaviours.^8^ Many research groups have since used the method (or variants of it) in various countries.^4^^,^^8^^,^^14^^,^^27^^–^^34^ The findings demonstrate that even in countries that meet or exceed the WHO’s population-level distribution targets, many people who inject drugs may have insufficient coverage at the individual level.^27^^,^^28^ Australia is often estimated to have one of the highest population-level coverage rates globally,^10^ supplying needles and syringes via an unlimited and unrestricted dispensing policy.^35^ However, at the individual level, estimates of the prevalence of insufficient syringe coverage range from 16% (117 out of 735 respondents) to 37% (133 out of 357 respondents).^27^^–^^30^ The ability, then, for individuals to achieve sufficient coverage may not be enhanced by increasing population-level syringe distribution alone. Instead, coverage may be best improved via incremental, targeted efforts which identify and respond to the unique social and individual contexts of people who inject drugs. The multiple international studies referenced above have already made substantial progress in this identification process. Numerous factors have been associated with insufficient coverage, such as homelessness,^36^ receptive syringe sharing,^8^^,^^32^ HCV positivity^31^ and personal syringe re-use.^4^^,^^8^^,^^30^^,^^32^^,^^37^ Conversely, studies have shown that those using needle and syringe programmes as a primary source of syringe acquisition, rather than via pharmacies or peers, and those currently receiving opioid substitution therapy, have lower odds of receiving insufficient coverage.^29^^–^^31^ This finding indicates how coverage interacts across harm reduction services, such as opioid substitution therapy,^2^ an insight which population-level measures are unable to provide. This research facilitates the tailoring of more efficient harm reduction services.

Population-level coverage is an indispensable element in needle and syringe programme evaluation, not least because its measurement methods are easily implemented and are recommended by WHO, UNODC and UNAIDS. However, coverage at the individual level broadens the understanding of harm reduction service delivery and performance, enabling differences across geographical areas and populations to be identified. The potential causes of these differences can then be investigated, thereby addressing some of the limitations^25^^,^^26^^,^^31^ of population-level coverage measurement. Consequently, we propose individual-level measurement as a complement, not a replacement, to current recommended population-level measures, thereby enhancing existing monitoring efforts and the planning of prospective services using practices based on context-specific evidence.^2^

## Implementation challenges

In measuring population-level coverage, passive data collection methods can be used. The key difficulty in measuring individual-level coverage is the need for active, primary data collection among samples large enough to generate meaningful results. This need can be met as a single standalone measurement of individual-level coverage or as routine coverage monitoring within established services. Both methods of data collection have challenges, but neither method must be too great a burden on services.

A single effort to measure coverage requires research resources that may be unused or unfamiliar to services. The staff of needle exchange services or academic research assistants need to be trained, or already knowledgeable, in confidential and ethical data collection methods, and in calculating individual-level coverage. Accurate data on the constituent parts of the coverage formula are essential for making meaningful estimates of individual-level coverage. Survey questions need to be developed and participants recruited. While these activities may be new to staff, essentially this is no different from other public health research that uses the expertise of existing personnel. Similar research was conducted in Myanmar, recruiting 512 people who inject drugs from five needle and syringe programmes across three geographical regions.^4^ The study involved the training of needle and syringe programme staff in the recruitment of people who inject drugs, at both needle and syringe programmes and during outreach, and the delivery of a confidential questionnaire. Training was delivered in two days and recruitment was completed in three months. Most importantly, this study was conducted at very low cost, with a low burden on needle and syringe programme staff. The coverage questionnaire took approximately 15 minutes to administer, including taking informed consent, and recruitment of respondents was incorporated into the general duties of the staff. While the ability to generalize results from specific geographical locations to the whole country is limited, the fact that the study was conducted across three locations with different characteristics provides some insight into national service delivery. For example, Mandalay has many illicit shooting galleries (informal, private locations where individuals can buy and inject drugs), while Yangon has none. Similar research has been conducted in both high-income^27^^,^^29^^,^^30^^,^^34^^,^^38^ and low-income settings.^32^^,^^33^ The Australian needle and syringe programme survey, conducted since 1995, follows a similar method. Programme staff annually recruit over 2200 needle and syringe programme presenting people who inject drugs.^39^ A recent version of the questionnaire included the necessary questions to calculate individual-level coverage.^30^

Alternatively, the necessary data can be collected as part of routine monitoring of client visits to needle and syringe programmes. Recording data at visits is already a component of some programmes,^18^^,^^40^ so the relevant questions can be easily absorbed into existing data collection efforts with minimal impact on staff or clients. To estimate coverage, a single time point can then be selected, such as the beginning of the month, to avoid double counting repeat client visits. In some Australian jurisdictions, needle and syringe programme data include questions to clients about their demographic profile and drug preferences.^40^ Additional questions about injection risk behaviour (e.g. syringe sharing) could be included and compared against coverage levels, providing an indication of risk profiles, albeit less detailed than in the multiple, international primary research studies described above. While routine data collection can be more difficult in some needle and syringe programmes (e.g. mobile programmes), ways can be found to facilitate data collection (e.g. using tablet computers). Also, some drug users will not wish to provide personal data while acquiring needles and syringes. These issues would no doubt have affected the examples of data collection mentioned above, with meaningful results produced nonetheless. The potential of regular individual-level coverage monitoring is currently being realized, with the Department of Health of the United Kingdom of Great Britain and Northern Ireland now reporting individual-level coverage as a routine indicator of HCV prevention activities.^41^

The challenges of implementation, however, extend beyond the practicalities of data collection. For example, individual-level coverage can only describe coverage among the specific group of users being surveyed, and cannot give the overall estimates provided by population-level coverage calculations. For this reason, we propose the measure as a complementary, rather than a replacement, method. The recruitment of a primary research sample requires a sample size large enough to make statistically valid findings. Effort is similarly needed to recruit a broad sample of sub-populations, for example, individuals who acquire syringes from pharmacies. Novel recruitment strategies, such as respondent-driven sampling^42^ or recruitment via locations other than needle and syringe programmes may assist with attaining a more representative sample. The measure is also subject to the common limitations in primary research, such as recall and social desirability bias. These are, however, standard issues of research bias. Existing limitations to individual-level coverage measurement also need to be addressed. For instance, a standardized method of measurement is yet to be established. Several variants of the original 2007 method^8^ have been used. One study^30^ specified syringe acquisition from multiple sources (as opposed to acquisition specifically from needle and syringe programmes). This solution partially addressed the acknowledged limitation of the WHO, UNODC and UNAIDS indicator’s inability to consistently capture pharmacy sales of needles and syringes. Another study in Australia^28^ showed that including additional parameters within the coverage formula (specifically, syringe stockpiling) changed coverage estimates considerably. The inconsistency in measurement methods limits the ability to compare different individual-level coverage measurements. Additional research has aimed to determine a standard individual-level measure,^37^^,^^43^ but more work is needed to decide on the optimal set of parameters and the timeframe for coverage calculation. For the present, we recommend the 2016 syringe stockpiling inclusive method of measuring past-month individual-level coverage.^28^

Finally, while population-level coverage measurements have specified targets, no such benchmarks exist for individual-level coverage measures. A target of 100% of people who inject drugs having at least 100% needle and syringe coverage is ideal, but is unlikely to be feasible given the pervasive barriers to achieving sufficient coverage. Therefore, setting prevalence targets for sufficient coverage may be unnecessary. Instead, individual-level coverage measurement should be used to monitor the quality of service delivery and identify sub-groups of people who inject drugs in need of tailored, targeted intervention.

## Conclusion

The issue of the existing focus on service provision, rather than behaviour, in current syringe coverage measurement has been discussed before.^2^ Current population-level coverage measures do not allow for incorporation of indicators of risk behaviour that can affect coverage substantially, and often miss key components of coverage, such as pharmacy sales. Measurement of coverage at the individual level represents the best available method of filling these vital knowledge gaps in the evaluation of harm reduction interventions. With this additional information, tailored and responsive harm reduction programmes can be created, specific to the localized and dynamic contexts within, which injecting drug use occurs. For individual-level measurement to be used consistently, we advise including the 2016 syringe stockpiling inclusive method^28^ of measuring individual-level coverage within standard WHO, UNODC and UNAIDS reporting packages.
